# Remote sensing approach in evaluating anthropogenic impacts on the spatiotemporal changes in net primary productivity of the Niger river basin, from 2000 to 2020^☆^

**DOI:** 10.1016/j.heliyon.2023.e21246

**Published:** 2023-10-24

**Authors:** Ogbue Chukwuka, Igboeli Emeka, Yahaya Ibrahim, Yeneayehu Fenetahun, You Yuan, Wang Yongdong

**Affiliations:** a National Engineering Technology Research Center for Desert and Oasis Ecological Construction, Xinjiang Institute of Ecology and Geography, Chinese Academy of Sciences, 818 South Beijing Road Urumqi 830011, Xinjiang, China; b University of Chinese Academy of Sciences, Beijing 100049, China

**Keywords:** CASA model, Net primary productivity, Anthropogenic activities, Niger river basin

## Abstract

Deterioration of the environment can be examined by utilizing a statistical evaluation of the effects of anthropogenic activities (beneficial or detrimental) on net primary productivity. The Niger River Basin's net primary productivity is significant both theoretically and practically for the management of the natural environment. It is important for her member countries to understand vegetation dynamics, maintain carbon balance, and ensure food security in the region. The research applied remote sensing to determine the relative impact of human activities on the net primary productivity of the Niger River Basin from 2000 to 2020. The study simulated the actual and potential net primary productivity using the Carnegie Ames Stanford Approach and Thornthwaite's Memorial Model respectively, while the result of the simulations was used to calculate human-influenced net primary productivity. The slope of the three simulations was calculated and merged in several scenarios using ArcGIS 10.8 to determine the impact of human activities on net primary productivity of the study area. The negative impacts of human activities were recorded in 89.88 % of the investigated area, while 10.12 % of the NRB had signs of positive impacts. Amongst the biomes, urban areas and bare land experienced the largest negative impacts (97.2 % and 99.8 %, respectively). The study advised the effectiveness of ecological restoration programs, through sound scientific and technical methods, such as those used in rural development, nomadic herding, environmental protection, and natural resource management policies.

## 1 Introduction

The world's vegetation is widely dispersed and essential to the global ecological cycle of water, material-energy distribution, and carbon cycle [[1], [2]]. Additionally, vegetation is essential for protecting the stability of ecosystems, soil and water conservation, and preserving the weather or climate [3], while observing land use cover changes on different geographical levels could essentially characterize the nature of the human-environment relationship [4]. Land use cover changes (LUCC) are observable dynamics in the physical cover of the surface of the earth, which includes water, land, plant, and man-made landscapes [5]. The spatiotemporal distribution characteristics of dynamics in land use and net primary productivity have become crucial for eco-environmental evaluation [6]. Net primary productivity (NPP) is the rate at which organic matter is accumulated by vegetation, which is equivalent to the variance between the amount of carbon received during photosynthesis and the amount of carbon used during plant respiration[[7], [8]]. Vegetation dynamics measured by NPP are prominent indicators of land deterioration and represent the biological mechanisms at play in either land degradation or restoration [9]. Direct and indirect impacts of LUCC on the environment especially on vegetation cover are unequivocal, with respect to their implications for global climate change. Human activity has generated major land degradation and ecological environmental issues due to the removal of vegetation and incorrect land use [10]. The impact of human activities has been extensively recorded by changes in the vegetation [11], and thus poses a serious threat to the preservation of biodiversity [12].

In Africa, a large population directly depends on the available natural vegetation for essential commodities such as fruits, wood for cooking, food, construction materials, and medicines made from herbs [13]. The health of the Niger River Basin (NRB) is essential for the ecosystem and West Africa's inhabitants. It is the major source of water and agricultural land to the member countries, providing food in terms of land and water resources. The NRB is under threat from the continued depletion of forest resources, which will eventually result in desert encroachment, as a result of the incorporation of her member nations into a global market economy. A process that has led to foreign investment in the extraction and lumber industries, which has significantly increased the rate of forest loss. Similarly, insufficient management of environmental resources and the NRB's expanding population have increased the need for food products in the face of food insecurity brought on by political instability and conflicts in the basin, such as in Nigeria and Niger. The detrimental impact of human activities leads to significant ecosystem changes in essential river basins [14], this necessitated the purpose and significance of our study which gears towards safeguarding the NRB from damaging human activities that can lead to the depletion of her natural resources, particularly the forest and water resources. A healthy forest serves to combat climate change, regulates carbon levels in the atmosphere, and maintains the global climate.

Earlier studies have shown that a variety of techniques have been employed to evaluate how human variables affect NPP. It includes all but correlation analysis [15], regression analysis [[16], [17], [18], [19]], Principal Component Analysis [20], sensitivity analysis [21], and Mann–Kendall trend analysis [[22], [23]], to mention but a few. Although these methods are employed in vegetation NPP studies, scientific research has the ability to be reproduced even in different study areas, hence, we adopted the Mann-Kendall analysis for our research. Given that, no in-depth study with respect to anthropogenic impact on NPP has been conducted in the NRB, our study aims at filling the academic gap by focusing on estimating the NPP of the NRB, and decoupling the impact of human influence on NPP, using remote sensing techniques. Our main objectives were to: (i) Estimate the actual, potential and human induced net primary productive; (ii) analyze the spatial pattern of NRB land cover and NPP changes from 2000 to 2020; (ii) calculate the slope of the simulated NPP (iii) quantify the changes in NPP induced human activities in the study area.

Therefore, this research conducted an analysis of the effect of human activities on the NPP of NRB between 2000 and 2020. To achieve this fit, the study simulated three forms of NPP, which include, Actual net primary productivity (ANPP), potential net primary productivity (PNPP), and human-influenced net primary productivity (HIPP). The slope of the estimated ANPP, PNPP, and HIPP, were extracted using the Mann-Kendall analysis of trends, and utilized to decouple and determine the relative influence of anthropogenic imprint on the NPP of the NRB. The conventional field measurements have been employed with success for small-scale observations with reliable NPP output[[24], [25]], but they are frequently tedious and exhausting [26], also because of the sparse measurement network, the methodologies are difficult to apply to the NPP estimation on broad scales such as the NRB. The study utilized robust remote sensing techniques which provides an accessible way to acquire satellite images collected over time with a high degree of temporal and spatial accuracy across a wide research area [27]. The findings of this study will not only give fundamental information for a thorough assessment of the vegetation NPP, but also to assist policymakers in understanding the condition of the vegetation in the Niger River Basin and developing future ecological policies pertaining to it.

## 2 Materials and methods

### 2.1 The study area

**Fig. 1:**
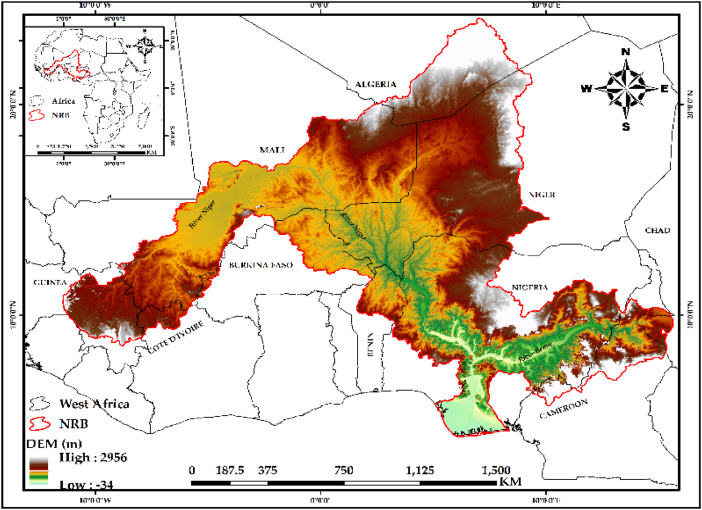
The Niger river basin (NRB).

The Niger River Basin (NRB) (Fig. 1), is Western Africa's largest river basin, situated between latitudes 5° and 23° N and longitudes 12° and 17° E [28]. It contains the Niger River, the 3rd longest river in Africa and the 9th longest river overall, which rises in the mountains of Guinea and empties into the sea through its delta in southern Nigeria.

More than 130 million people live within the active basin of the NRB, which occupies 2.13 million km^2^ total area and used together by nine nations: Cameroon, Burkina Faso, Ivory Coast, Benin, Mali, Chad, Nigeria, Guinea, and Niger [29]. The bulk of the member countries, which experience significant urbanization and population growth (projected annual average of 3.2 %), face a fragile environment characterized by political upheaval, insecurity, and difficult climate conditions [29]. The waters of the Niger River and its associated streams are an important resource that provides the member countries with a significant amount of water for transportation, drinking, industry, agriculture, and energy [30]. The annual rainfall differs from more than 4000 mm in southern Nigeria and Cameroon to fewer than 400 mm (and often none at all) on the Sahara Desert's borders in northern Mali and Niger. Climatic zones range from hyper-arid to sub-equatorial [31]. The largest sector in the basin, agriculture, employs 80 % of the workforce, yet due to erratic rainfall and rapidly shifting river flows, about 3/4 of the population experiences food insecurity. Subsistence farming accounts for about 78 % of overall production, with fishing accounting for 1–4%, crops 25–35 %, and livestock 10–15 % [30,32]. Cassava, yams, groundnuts, rice, millet, plantains, sorghum, maize, cocoa beans, cotton, and sugar cane are all the main crops found in the basin [31].

### 2.2 Methods

#### 2.2.1 Data collection

The Java-scripted Google Earth Engine (GEE) played a vital role in providing the database and interface for acquiring and preprocessing a great deal of Satellite data, which represents the most comprehensive archive of Earth's observations to date. These data include.

1. 300 m resolution Digital Elevation Model (DEM) of the study area from Shuttle Radar Topography Mission (SRTM).
2. The Terra-Climate dataset provided the solar radiation, actual evapotranspiration (AET), precipitation, and potential evapotranspiration (PET) dataset.
3. MOD13A1 provided the Normalized Difference Vegetation Index (NDVI)
4. The MODIS/061/MOD11A1 provided the surface temperature dataset.
5. The land cover map was obtained from the European Space Agency's Climate Change Initiative (ESA-CCI) database.
6. Population data of the member countries were acquired from the Food and Agricultural Organization (FAO).

The summary of the data is represented in Table 1.

**Table 1:** Data sources and basic characteristics.

Dataset Category	Dataset Source	Data Processing Interface	Study Year	Spatial resolution(meters)
Solar Radiation	TerraClimate	GEE	2000–2020	4638.3
Potential evapotranspiration	TerraClimate	GEE	2000–2020	4638.3
evapotranspiration	TerraClimate	GEE	2000–2020	4638.3
Precipitation	TerraClimate	GEE	2000–2020	4638.3
NDVI	MODIS/061/MOD13A1	GEE	2000–2020	500
Surface temperature	MODIS/061/MOD11A1.	GEE	2000–2020	1000
Land Cover	ESA CCI	ARCMAP 10.8	2000–2020	300

#### 2.2.2 Data processing and analysis

The data generated for this study were processed and analyzed using the Google Earth Engine online platform, Python 3.11, and ArcGIS 10.8. The actual, potential, and human-induced net primary productivity all measured in gC/m^2^/y-^1^ for this study, are a prerequisite for calculating the trends and slopes of the net primary productivity. The slopes measured in degrees are pertinent for analyzing human impacts on net primary productivity of the Niger River Basin.

#### 2.2.3 Estimation of actual net primary productivity (ANPP)

The Carnegie Ames Stanford Approach (CASA) model has been extensively utilized for inversion simulations of carbon sequestration, simulation of agricultural land net primary productivity (NPP), and proven effective at capturing spatial and temporal NPP dynamics [33]. The CASA model is a light use efficiency model based on satellite remote sensing (Zhu et al., 2007). The actual net primary productivity (ANPP) is calculated using the amount of absorbed photosynthetically active radiation (APAR) by the vegetation and the actual light utilization efficiency (ε) which measures how effectively that radiation results in an increase in plant biomass. The model was calculated for the study period between 2000 and 2020 using a combination of codes in Python 3.11. The purpose of this model is to simulate the actual vegetation net primary productivity of the study area. Input data required for the calculation include; solar radiation (W/m^2^), potential evapotranspiration (mm), normalized different vegetation index, temperature (^0^C), actual evapotranspiration (mm) and land cover map (2000–2020). The formula [34] is given as:

(1) NPP(x,t)=APAR(x,t)×ε(x,t)

(2) APAR(x,t)=SOL(x,t)×FPAR(x,t)×0.5

NDVI(x,t)‐NDVIi,min

(3) $$\text{NDVIi,}{\text{ma}}_{x\;}‐\;{\text{NDVI}}_{i,\min}\text{AR}\left(x,t\right)=x\;\left({\text{FPAR}}_{\max}-{\text{FPAR}}_{\min}\right)+{\text{FPAR}}_{\text{mi}}n$$

Where; NPP(*x, t*) is the net primary productivity (gC/m^2^/y-^1^) of pixel *x* in month *t*, FPAR(*x, t*) is fraction of absorbed photosynthetically active radiation of pixel *x* in month *t*, (no unit). To estimate FPAR for different types of vegetation, it requires the highest and minimum NDVI values for the type of vegetation and the related maximum and minimum FPAR values. SOL(*x, t*) is the solar radiation of pixel *x* in month *t*, (W/m^2^). APAR(*x, t*) is the absorption of photosynthetically active radiation (MJ m^2^) of pixel *x* in month *t*. The APAR is computed using FPAR and SOL. The values of NDVI_max_, NDVI_min_, and light energy usage efficiency are calculated for various land cover types using the improved results of the prior investigations [35]. 0.5 % is the constant ratio of the solar radiation used by vegetation and for estimating photosynthetically active radiation. The light use efficiency ε(*x, t*) of pixel *x* in month *t* is determined as follows:

(4) $$\varepsilon =T_{\varepsilon 1}\times T_{\varepsilon 2}\times W_{\varepsilon }\times \varepsilon_{\max}$$

Where; (ε) is the light use efficiency, T_ε_ – temperature stress and W_ε_ is water stress efficiency. T_ε1_ and T_ε2_ represent the impacts of high and low temperature stress, respectively. The highest achievable light usage efficiency is represented by ε while W_ε_ represents the effects of water stress.

#### 2.2.4 Simulation of potential net primary productivity (PNPP)

The creation of the Thornthwaite Memorial model was based on the amendment of data used in the Miami model, to include the potential evapotranspiration, precipitation and temperature in estimating potential net primary productivity (PNPP). This model was simulated for the study period of 2000–2020 through a combination of java codes in Google Earth Engine online platform. The Thornthwaite simulation for PNPP is determined as follows:

(5) $$L=3000+25t+0.05t^{3}$$

(6) $$V=\frac{1.05r}{}$$

(7) $${\sqrt{1+\left(1+1.05r/l\right)}}^{2}$$

$$\text{PNPP}=3000\left[1-e^{-0.0009695\left(v-20\right)}\right]$$

Where; PNPP represents potential net primary productivity, t represents the annual average temperature (0 °C), L is the mean annual potential evapotranspiration (mm), r is the mean annual precipitation (mm), and v is the yearly average actual evapotranspiration (mm).

#### 2.2.5 Simulation of human-influenced net primary production

Human-influenced net primary production (HIPP) is the disparity between PNPP and ANPP. It determines how much NPP is lost or increased as a result of human intervention and how this affects the productivity of vegetation. For the purpose of calculating HIPP, the potential net primary productivity is denoted as NPP_pot_, while the actual net primary productivity is denoted as NPP_act_. The formula is adopted from Li et al., 2016 [36], and stated below;

(8) $$\text{HIPP}={\text{NPP}}_{\text{pot}\left(x,t\right)}\;─\;{\text{NPP}}_{\text{act}\left(x,t\right)}$$

Where; NPP_act_ and NPP_pot_ denotes actual and potential NPP, in the x-space and t-time of the study area. Whereas a negative value of HIPP indicates an increase in NPP caused by human activity, a positive HIPP indicates a decrease in NPP caused by human activity.

The next section encompasses the trend and slope calculation of the simulated net primary productivity, in order to further determine the impact of human activities on NPP of the Niger River Basin, using the Mann-Kendall trend analysis.

#### 2.2.6 Trend and slope analysis

In this study, the Mann-Kendall trend analysis and Theil-Sen's slope were employed to determine the trend and slope analysis of net primary productivity in the NRB. The advantage of Mann-Kendall test is that the distribution may be simply calculated from the time series, necessitating no additional data or simulations, and the computation is much quicker than that of the parametric tests. β is a non-normalized parameter, it can only indicate how much the time series' trend has changed overall, making it impossible to determine whether the trend change is significant on its own. Consequently, the Mann-Kendall approach has to be used in conjunction with the trend's significance test.

Theil-Sen's slope is frequently used in time-series studies of vegetation at the pixel scale since it doesn't require the data to fit a particular distribution model or be affected by outliers. The calculation was carried out in Python 3.11 interface and the formula as adopted from Ref. [37], for Sen's slope is as follows:

(9) $$\beta =\text{median}\frac{\text{xj}-\text{xi}}{j-i},\forall j\;>\;i$$

When; β is the slope, xj and xi are the NPP data of two periods, β > 0, an increase in the time series data's change trend, and β < 0, indicates a decrease in the change trend. For testing, the Mann-Kendall test creates statistical variables S for time series data from x_1_ to x_n_. The formula (from 11 to 14) as adopted from Ref. [37] is given as;

(10) $$S=\sum_{i=1}^{n-1}\sum_{j=i+1}^{n}\text{sgn}\left(x_{j}-x_{i}\right)$$

(11) $$\text{sgn}\left(x_{j}-x_{i}\right)=\left\{ \begin{array}{c} 1\;\text{if}\left(x_{j}-x_{i}>0\right)\\ 0\;\text{if}\left(x_{j}-x_{i}=0\right)\\ -1\;\text{if}\left(x_{j}-x_{i}<0\right) \end{array}\right.$$

The Variance formular is as follows;

(12) $$\text{Var}\left(S\right)=\frac{n\left(n-1\right)\left(2n+5\right)-\sum_{i=1}^{m}t_{i}\left(t_{i}-1\right)\left(2t_{i}+5\right)}{18}$$

Where Var is the variance, t_i_ is the number of recurrent data points in the ith group of repeated data, m is the number of reoccuring data groups in the time series, sgn is a sign function, n is the length of the time series while S is the test statistics. The formula for calculating Z when n ≥ 10 is:

(13) $$Z=\left\{ \begin{array}{c} \frac{S-1}{\sqrt{\text{var}\left(s\right)}}\;S>0\\ 0\;S=0\\ \frac{S+1}{\sqrt{\text{var}\left(s\right)}}\;S<0 \end{array}\right.$$

In this study, the time series under inquiry covered a period of 21 years, hence the trend was tested using the test statistic Z with at α = 0.05 level of confidence. When β is higher than zero, a positive trend occurs, while if the value of β is lower than zero, a negative trend occurs. If Z's standard value is larger than 1.96, the trend significance test has been passed.

The next section addressed the combined scenario of the calculated slopes to determine the anthropogenic impact on net primary productivity of the NRB.

#### 2.2.7 Slope scenario assessment

The study carried out a quantitative assessment to decouple the proportionate relevance of anthropogenic activities affecting the net primary productivity (NPP) of the study area. The slopes of potential net primary productivity (S_PN_), actual net primary productivity (S_AN_), and Human-influenced net primary productivity (S_HN_) were calculated, using the ArcGIS 10.8 raster calculator in Map Algebra section of the arc toolbox. A positive slope for S_AN_ from 2000 to 2020 in the study area represents increased NPP, whereas a negative S_AN_ represents a reduction in NPP. When S_HN_ is negative, human activities boost NPP and encourage vegetation reconstruction whereas when S_HN_ is positive, these activities have a deteriorating effect on NPP, causing it to decline in value. Therefore, in decoupling the relative roles of climate change from human influence, the study classified six scenarios emanating from the mitigating effect of positive S_AN_ and deterioration effect when S_AN_ is negative. The restoration effect refers to any sort of activity or practice that brings about a positive impact on NPP of the study area. Human restoration activities for instance, imply human practices that protect and positively affect the vegetation of the environment. The deterioration effects are negative practices that harm the environment's vegetation net primary productivity. These scenarios were adopted and modified from Ref. [36] stated below; For Positive S_AN_ Scenarios.

1. S_PN_ < 0, S_HN_ < 0: Mitigating impact of human activity on NPP.
2. S_PN_ > 0, S_HN_ > 0: Mitigating impact of climate on NPP.
3. S_PN_ > 0, S_HN_ <0: Combined mitigating impact of anthropogenic activity and climate on NPP.
  For Negative S_AN_ Scenarios.
1. S_PN_ > 0, S_HN_ >0: Negative impact of human activities on NPP
2. S_PN_ < 0, S_HN_ <0: Negative impact of climate change on NPP
3. S_PN_ < 0, S_HN_ >0: Combined negative impact of anthropogenic activity and climate on NPP

The summary of the scenarios, given in Table 2, below.

**Table 2:** Six different scenarios of phenomenal impacts on the Net primary productivity of the Niger River Basin.

NPP status	Scenario	S_AN_	S_PN_	S_HN_	Classification
NPP Restoration	Scenario 1	>0	<0	<0	Human
	Scenario 2	>0	>0	>0	Climate
	Scenario 3	>0	>0	<0	Human and Climate
NPP Deterioration	Scenario 1	<0	>0	>0	Human
	Scenario 2	<0	<0	<0	Climate
	Scenario 3	<0	<0	>0	Human and Climate

S_AN_ = Slope of Actual Net Primary Productivity; S_PN_ = Slope of Potential Net Primary Productivity; S_HN_ = Slope of Human influenced Net Primary Productivity; NPP = Net Primary Productivity.

## 3 Result

### 3.1 Spatial distribution of NPP simulations in the NRB

**Fig. 2:**
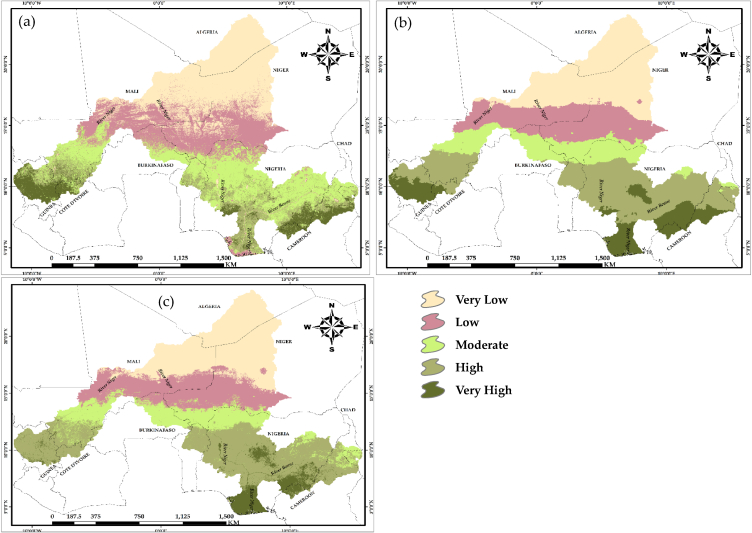
Simulated net primary productivity (NPP): (a) Actual NPP; (b) potential NPP; (c) human influenced NPP.

The mean pixel actual net primary productivity (ANPP) values estimated from 2000 to 2020, totaled 1353gC/m^2^/yr^−1^, is equivalent to 349.20gC/m^2^/yr^−1^. The ANPP of the NRB decreased in a south-to-north direction. At the farthest north of the arid Sahelian borders of the Niger River Basin (NRB), the ANPP is very low and in the central area, it varied between low and moderate values, while towards the southern edge of the NRB, it increased between high and very high values. ANPP levels decreased toward Mali, Niger, the northern part of Nigeria, and a portion of Chad, but the regions with substantially greater ANPP are throughout the southern part of Nigeria, northeastern Benin, northwestern Cote d'Ivoire, northeastern Guinea, and a portion of Cameroon (Fig. 2a).

Similarly, the potential net primary productivity (PNPP) with an average value of 1227 gC/m^2^/yr^−1^ and totaling 2850gC/m^2^/yr^−1^, decreased from the south towards the north region of the NRB (Fig. 2b). It was very low in the northernmost part, low to moderate at the center while high and very high at the southern part. The largest PNPP occurred in the south, southeast, and southwest of the NRB because natural productivity is highest in the tropics. Likewise, the mean human-influenced net primary productivity (HIPP) values estimated at pixel values from 2000 to 2020 and totaled 2,774gC/m^2^/yr^−1^, is equivalent to 883.62gC/m^2^/yr^−1^. The HIPP in (Fig. 2c), also decreased in a south-to-north direction in the NRB. These spatial distributions coincide with the distribution of land cover classes across the study region. Areas with favorable climate such as the tropical rainforest in the southernmost portion of the research area is high in NPP compared to the arid regions towards the north. Table 3, displays the area and percentage of each land cover type in the NRB in 2020.

**Table 3:** Area and proportion of land cover types in the Niger River Basin in 2020.

	Bare Land	Crop Land	Forest	Grass Land	Sparse Vegetation	Urban Area	Water Body	Wet Land
Km^2^	571306.3	627660.7	417380.7	415533.7	147147.7	5153.85	15675.36	11686.96
%	25.83	28.38	18.87	18.79	6.65	0.23	0.71	0.53

Km^2^ – Kilometer Square, % – Percentage.

### 3.2 Variation of ANPP in different LUCC classes

**Fig. 3:**
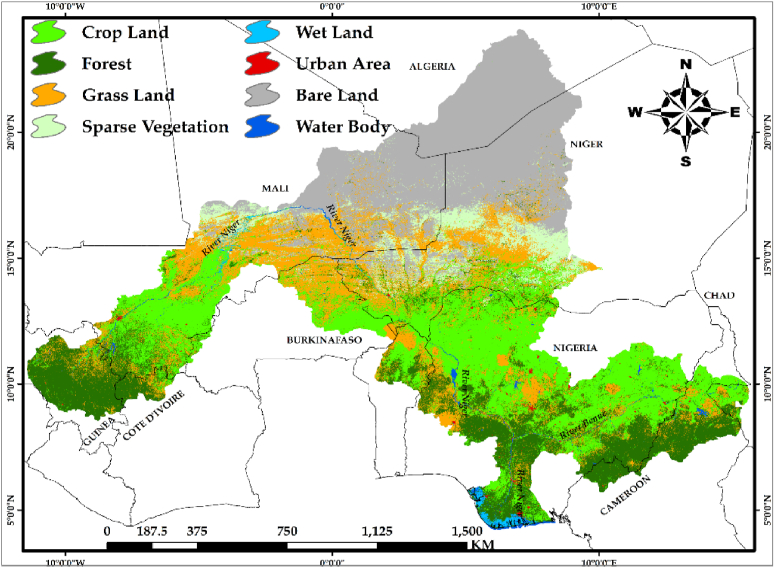
Land use cover change map of the Niger river basin.

An analysis of mean actual net primary productivity (ANPP) with respect to dynamics in land cover types reveals substantial variations in NPP across the identified land cover classes in the study area. Fig. 3, shows the land cover classes identified in the NRB.

**Fig. 4:**
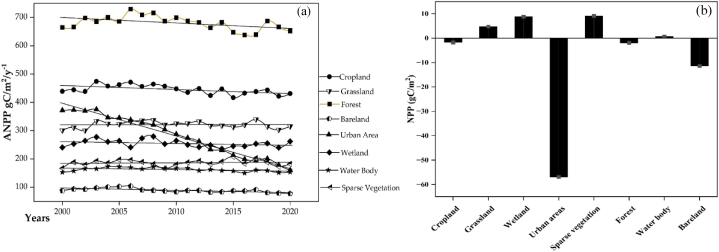
Statistic of net primary productivity (NPP) In the study area: (a) Inter-annual average NPP values In each Biome from 2000 to 2020; (b) observed NPP changes In each Biome.

The urban area exhibits the highest reduction (−211.29gC/m^2^/yr^−1^) by 56 %, covering the area of 4904.98 km^2^. The investigation uncovered further areas of negative changes in NPP, such as in forest areas (−13.01gC/m^2^/yr^−1^) and bare lands (−9.99gC/m^2^/yr^−1^) at 1.96 % and 11.37 % respectively. The study also revealed a positive change in wetlands (21.17gC/m^2^), water bodies (0.95gC/m^2^/yr^−1^), and sparse vegetation (15.23gC/m^2^/yr^−1^) at 8.79 %, 0.62 %, and 9.13 % respectively, while the wetlands and places with sparse vegetation recorded the most increase in NPP, with respective areas of 11378.7 km^2^ and 1427.54 km^2^. Fig. 4a, shows average NPP values from 2000 to 2020 for the different LUCC class types represented in the study area while Fig. 4b, depicts the positive and negative variations in NPP of the various LUCC classes.

### 3.3 Trends in human-influenced net primary productivity (HIPP)

**Fig. 5:**
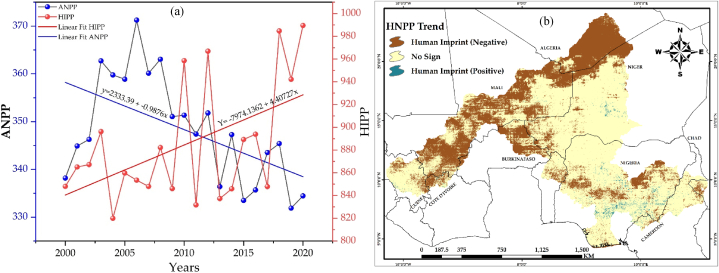
Trends in Net Primary Productivity (NPP) in the Study Area: (a) Linear Changes in Values of Actual NPP and Human influenced NPP from 2000 to 2020; (b) Spatial Trend Changes in Human influenced NPP from 2000 to 2020.

The study examined trends in human influenced net primary productivity (HIPP) from 2000 to 2020 using Python 3.11 platform to assess the impact of anthropogenic actions on the decreasing NPP (Fig. 5a). The mean HIPP values during the course of the 21-year research generally showed an upward trend, rising from 848.05gC/m^2^/y^−1^ in 2000 to 989.55gC/m^2^/y^−1^ by 2020. This indicates that the decline in NPP because of anthropogenic activities was evident in most areas as seen in Fig. 5b, of the Mann-Kendall analysis of trend of the HIPP across the NRB.

**Fig. 6:**
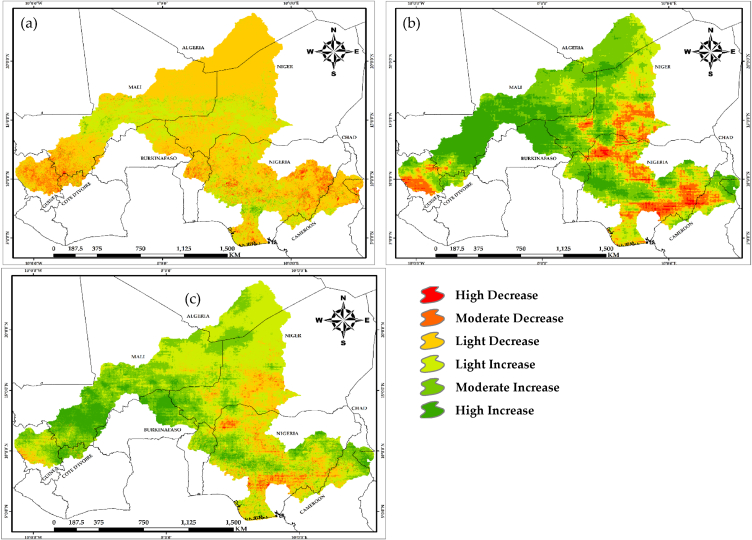
Slopes of net primary productivity (NPP) of the Niger river basin: (a) Actual NPP slope (S_AN_); (b) potential NPP slope (S_PN_); (c) human influenced NPP slope (S_HN_).

Furthermore, the slopes of actual net primary productivity (S_AN_), potential net primary productivity (S_PN_), and Human-influenced primary productivity (S_HN_) were extracted as shown in Fig. 6. The study focused on S_HN_ to determine areas of NPP decline and gains due to anthropogenic influence. The research revealed that 82.35 % (1818152.06 km^2^) of the total area of NRB was represented with S_HN_ > 0 (i.e., adverse impacts of anthropogenic activity on vegetation), while the remaining 17.65 % (389774.70 km^2^) was made up of S_HN_ < 0 areas. Over human interference, NPP decreased as seen in the arid north, mixed portions cutting across Nigeria and Chad, northwest and southwest across Mali, Burkina-Faso, Guinea, and part of Cote d'Ivoire, while NPP increased in the northeastern part, southeast part across southern Niger, Nigeria, and Cameroon.

### 3.4 Unveiling the impact of anthropogenic presence on NPP

**Fig. 7:**
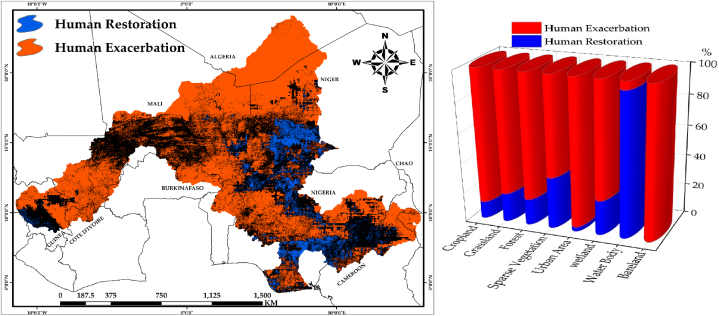
The Impact of Human Activities on the Net Primary Productivity (NPP) of the Study Area: (a) Trend of Human presence on NPP; (b) Proportions of Positive and Negative Impact of Human Activities on different Biomes.

This study focused on decoupling the impact of anthropogenic activities on net primary productivity (NPP) of the Niger River Basin (NRB). This was estimated using the trend analysis slope in a combination of different scenarios with reference to Table 2, to be able to decouple human impacts on the changing NPP of the NRB in (Fig. 7a). The study found that highly negative human impacts were present in the arid northern part of the NRB, a mixed portion running through Nigeria and Chad, the northwest and southwest parts cutting across Mali, Burkina Faso, Guinea, and a portion of Cote d'Ivoire, while the north-eastern part, southern Niger, Nigeria, and Cameroon recorded positive human impacts. Out of a 100 % assessment, restoration impact of human activities accounted for 10.12 % covering about 139640.85 km^2^ where anthropogenic activities had a net beneficial influence on NPP, while 89.88 % (1,240,091.88 km^2^) accounted for the negative impact of human activities. Thus, human activities played a key role in vegetation degradation in the NRB.

Fig. 7b shows the areas and percentages of the impact of human activities on land cover types of the study area. The study revealed that restoration impact of human activities occurred in 41687.43 km^2^ of cropland (11 %), 28985.1 km^2^ of grassland (19.16 %), 37196.4 km^2^ (17.4 %) of Forest area, 23472.94 km^2^ (34 %) of sparse vegetation, 84 km^2^ of Urban area at 2.8 %, 828.2192 km^2^ of wetland (23 %), 1321.274 km^2^ water body (23.2 %) and 0.2 % of bare land (783.52 km^2^). Analogously, cropland (326699.5 km^2^), grassland (122275.9 km^2^), forest (176897.8 km^2^), sparse vegetation (45200.1 km^2^), urban area (2904.76 km^2^), wetland (2763.797 km^2^), water body (76.82875 km^2^), and bare land (529088.1 km^2^), each accounted for negative impacts of human activities at the rate of 89 %, 80.84 %, 82.6 %, 65.8 %, 97.2 %, 77 %, 76.8 % and 99.8 % respectively.

This study estimated the ANPP, PNPP, and HIPP of the study area. The trend and slope analysis for the simulated NPP were also calculated. The resulting slopes were further combined in a series of scenarios to illustrate the human impact on the NPP of the NRB. The study discusses the results in the next section.

## 4 Discussion

The net primary productivity (NPP) of the Niger River Basin (NRB) is not uniformly distributed, with certain areas exhibiting higher values than others (Fig. 2). Anthropogenic activities entail deliberate modification of nature, which includes both productive activities and lifestyles [[38], [39]], and anthropogenic disturbances have gradually become the primary driving catalyst for the dynamics of the NRB's natural environment. This study, notwithstanding the difficulties in separating anthropogenic impacts on NPP however, presented an approach, which distinguished between two types of influences (human and climate), quantitatively and spatially, and identified regions where anthropogenic activity contributes to changes in NPP. To achieve this fit, both ANPP, PNPP and the HIPP was simulated to identify the impacts of human activities on net primary productivity of the NRB by combining the slope output of the above-mentioned simulations. Previous research such as [9,36,[40], [41], [42]] have also employed similar technique in decoupling anthropogenic impacts from climatic influence on NPP studies. Evidently, LUCC is perceived as a result of direct human impact on the environment [[43], [44]]. The decreasing NPP of the NRB as a result of human impact were due to over-harvesting of forests, overgrazing, over utilization of water resources which leads to water crisis, irrational reclamation of land for agricultural and the transformation of land cover types to accommodate other activities. The statistical result of the impact of humans on land cover revealed that, urban areas and bare land were most affected by the widespread exacerbation effects of human imprint across the NRB (97.2 % and 99.8 %, respectively). This implies that restoration impacts is lowest within the urban and bare land areas at 2.8 % and 0.2 %, respectively (Fig. 7b). This has serious implications in the study area in terms of urban and bare land expansion due to human activities, especially for bare areas, which could result to desert encroachment from the northern extent of the study area towards the southern portion. From Fig. 3, it is evident that the northern part of the study area is bare and dry, increased human activities could result in expansion of these bare areas towards the southern part, thereby leading to desert encroachment.

In comparison, our study coincided with the study conducted in Sudano-sahelian countries of Africa by Stéphenne and Lambin (2001) [45], which opined that deforestation has increased as a result of Sahel countries' efforts to address the energy needs of their large urban populations and to meet its demands. More so, the conversion of natural grasslands into farmlands for agricultural purposes in order to provide food for the growing population. In some cases, the attempt at land reclamation has led to negative outcomes such as the Boko Haram insurgency and cattle herders’ conflict with farmers in a quest to convert the arable lands into pastoral farming, such as in Nigeria. These conflicts are attributed to increasing human activities, such as deforestation, conversion of lands for agricultural practices, and increasing population that puts pressure on the available resources, which leads to urban and bare land expansion, as seen in Fig. 7b. It is pertinent to note that nomadic herding (cattle herders) is an occupation identified with the northern part of the study area, especially in Nigeria. While these negative human activities are going on, the bare land areas keep encroaching into the more vegetative southern part of the study area, given that the northern extent of NRB is characterized by arid lands with less vegetation cover. The cattle herders in a quest to feed their cattle continue to migrate south in search of greener pastures, thereby encroaching into the farmers' lands and destroying crops on their path, an action which eventually leads to serious crashes between these groups.

**Fig. 8:**
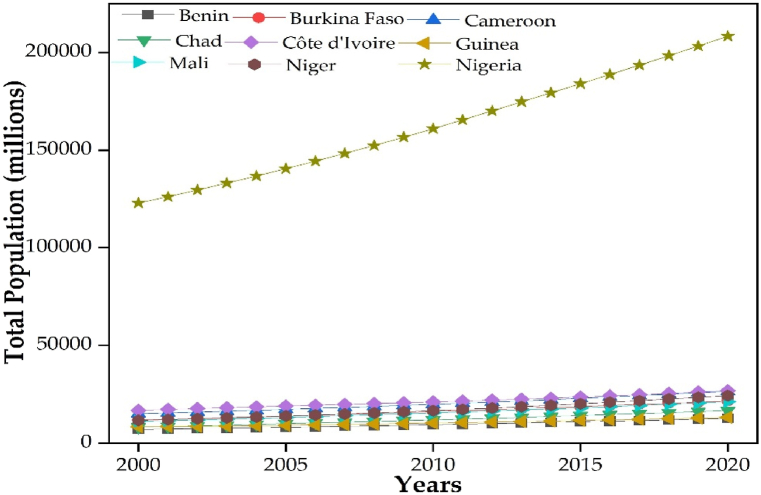
Total population of member countries of the Niger river basin.

In recent years, Nigeria's population has had an unprecedented pace of expansion (Fig. 8), and previous research has demonstrated that human activities like commercial logging, livestock, and pasture practices, have significantly affected land usage in Nigeria[[46], [47]].

The study coincides with Petrakis et al. (2017) in the view that these human-related variables have increased the population's need for agricultural activity intensity, land, and deforestation rate, which has resulted in a decrease in NPP [48] and further demonstrates the negative impacts of human on NPP. Our study also conforms to the findings of [37] in the conventional Lake Chad Basin research area, which emphasizes on the population pressure on land, intensity of agricultural activities and deforestation that distorts land cover of the area. Similarly, these human activities encourages the expansion of dry areas into regions that are not formally dry in the Niger River Basin.

This research demonstrated the dramatic impact of anthropogenic activities (positive and reversal) on the NPP of NRB, and assessing the time-dependent variation of terrestrial NPP. Its underlying human-influenced driving forces can aid in more efficient management and use of the planet's resources. As a result, we should implement and improve environmental protection measures aimed at protecting the NRB.

## 5 Conclusion

The study simulated the net primary productivity (NPP) of the Niger River Basin (NRB), using the Thornthwaite Memorial model and the CASA model from 2000 to 2020. Generally, the spatial distribution of NPP declined in a south-to-north direction in the study area. The study then quantified the relative contributions of anthropogenic activities to changes in the NRB's NPP by calculating the human-influenced net primary productivity and then, the slopes of the actual, potential, and human-induced net primary productivity. The negative impacts of human activities were recorded in 89.88 % of the investigated area, while 10.12 % of the NRB had signs of positive impacts of human activities. Amongst the land cover classes, urban areas and bare land experienced the largest negative impacts (97.2 % and 99.8 %, respectively), While restoration effects of human activities were seen in portions of Niger, Cameroon, and Guinea as well as the central part of Nigeria, anthropogenic activities led to the decrease of vegetation NPP in almost all part of the basin. The essence of this study is to provide reasons to protect the NPP of the study area. Depleted vegetation resources and over exploitation of other resources in the NRB by the present generation poses a serious threat for the future generations. Human interventions should be adequately monitored and controlled since they have a measurable effect on changes in the NPP of the NRB. Consequently, we must improve the efficiency of ecological restoration projects through sound scientific and technical methods, such as those used in rural development, nomadic herding, environmental protection, and natural resource management, for sustainable development. The Niger River Basin Authority, the body responsible for maintaining the health of the basin can criminalize incessant felling of trees without prior approval from the relevant authorities. The responsible government agencies may also introduce schemes that will employ the local people for protecting the forests. The study suggests for future research an evaluation of the strengths and weaknesses of existing environmental policies in the study area. More so, to investigate other factors affecting the NPP of the NRB, such as climatic factors.

The negative impact of human activities, revealed in the NRB would encourage desert encroachment towards the southern region of the study area and exacerbate human related crisis over struggle for available vegetation resources. Let us all join hands together to achieve the greening of our environment and stand together in fight against negative human activities that alters the environment and possibly leads to climate change.

## Data availability statement

The data used in the course of this research are shown in the manuscript and available from the author upon request. The data can also be found @ https://code.earthengine.google.com/c89d7ea6c13bf8b27c8e99c03fdbefb0.

## CRediT authorship contribution statement

**Ogbue Chukwuka:** Conceptualization, Methodology, Investigation, Writing – original draft. **Igboeli Emeka:** Formal analysis, Data curation. **Yahaya Ibrahim:** Data curation, Formal analysis. **Yeneayehu Fenetahun:** Investigation. **You Yuan:** Data curation, Investigation. **Wang Yongdong:** Data curation, Funding acquisition, Project administration.

## Declaration of competing interest

The authors declare the following financial interests/personal relationships which may be considered as potential competing interests.
